# Investigating Information Dynamics in Living Systems through the Structure and Function of Enzymes

**DOI:** 10.1371/journal.pone.0154867

**Published:** 2016-05-05

**Authors:** Robert Gatenby, B. Roy Frieden

**Affiliations:** 1 Departments of Integrated Mathematical Oncology and Radiology, Moffitt Cancer Center, Tampa, FL 33612, United States of America; 2 College of Optical Sciences, University of Arizona, Tucson, AZ 85721, United States of America; Russian Academy of Sciences, Institute for Biological Instrumentation, RUSSIAN FEDERATION

## Abstract

Enzymes are proteins that accelerate intracellular chemical reactions often by factors of 10^5^−10^12^*s*^−1^. We propose the structure and function of enzymes represent the thermodynamic expression of heritable information encoded in DNA with post-translational modifications that reflect intra- and extra-cellular environmental inputs. The 3 dimensional shape of the protein, determined by the genetically-specified amino acid sequence and post translational modifications, permits geometric interactions with substrate molecules traditionally described by the key-lock best fit model. Here we apply Kullback-Leibler (K-L) divergence as metric of this geometric “fit” and the information content of the interactions. When the K-L ‘distance’ between interspersed substrate *p*_*n*_ and enzyme *r*_*n*_ positions is *minimized*, the information state, reaction probability, and reaction rate are *maximized*. The latter obeys the Arrhenius equation, which we show can be derived from the geometrical principle of minimum K-L distance. The derivation is first limited to optimum substrate positions for fixed sets of enzyme positions. However, maximally improving the key/lock fit, called ‘induced fit,’ requires *both* sets of positions to be varied optimally. We demonstrate this permits and is maximally efficient if the key and lock particles *p*_*n*,_ *r*_*n*_ are quantum entangled because the level of entanglement obeys the same *minimized* value of the Kullback-Leibler distance that occurs when all *p*_*n*_ ≈ *r*_*n*_. This implies interchanges *p*_*n*_ ⇄ *br*_*n*_ randomly taking place during a reaction successively improves key/lock fits, reducing the activation energy *E*_*a*_ and increasing the reaction rate *k*. Our results demonstrate the summation of heritable and environmental information that determines the enzyme spatial configuration, by decreasing the K-L divergence, is converted to thermodynamic work by reducing *E*_*a*_ and increasing *k* of intracellular reactions. Macroscopically, enzyme information increases the order in living systems, similar to the Maxwell demon *gedanken*, by selectively accelerating specific reaction thus generating both spatial and temporal concentration gradients.

## Introduction

Living organisms, uniquely in nature, encode, propagate, and use information [1] to produce stable, highly-ordered structures that are also complex, dynamical, semi-open systems far from thermodynamic equilibrium. But, what is biological information and how is information used to maintain the ordered structure and function of a living system [2–5]? While it is apparent that information storage and process are fundamental characteristics of living systems, the principles governing information dynamics in biology remain unclear.

Enzymes are central to the function of living systems and facilitate the work necessary to maintain order [6]. Once synthesized as a string of amino acids specified by the nucleotide triplets in the gene, a protein is typically subjected to post-translational modification such as phosphorylation. Importantly, post translational modifications reflect temporally variations in the status of the cell (e.g. ATP concentrations [7]). Thus the 3 dimensional shape of the enzyme represents a summation of both heritable and current information within the cell. This composite information produces a 3 dimensional structure that is the low free-energy state for the amino acid sequence plus post-translational modifications. It will be seen that this minimum state represents, as well, one of minimum Kullback-Leibler divergence, i.e. maximal order, between substrate and enzyme codons. These effects result from a doubly-optimized lock and key interaction between substrate and enzyme codons.

**Fig 1:**
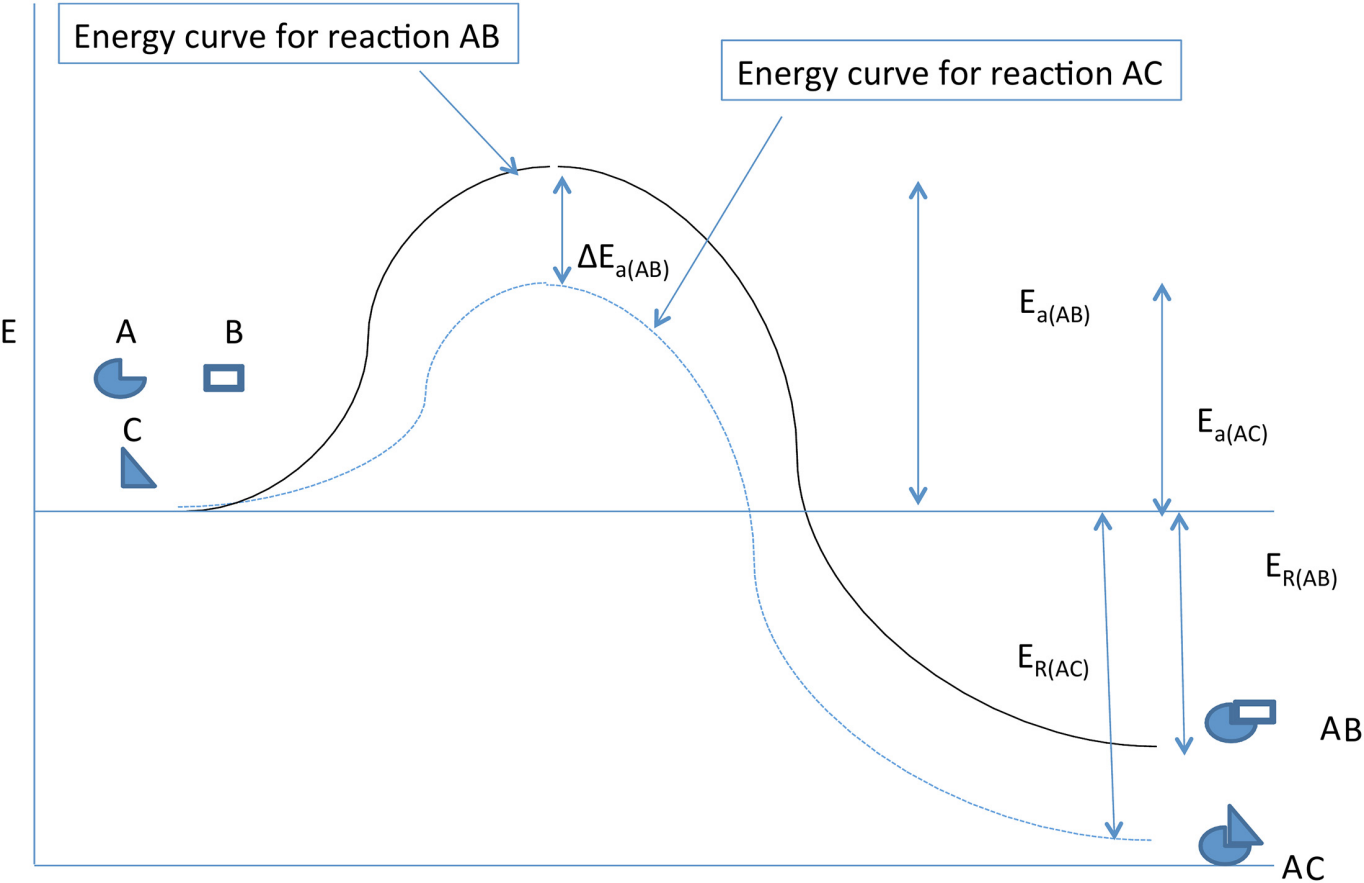
A simplified model of a reaction with and without an enzyme. Substrate B is yields products C and D with a release of free energy ER. Although the overall reaction is thermodynamically favorable, there is an energy barrier (the activation energy [Ea]) that decreases the rate of the reaction (k). The enzyme, through a key-lock geometric binding with the substrate, has a net effect of reducing the Ea and accelerating the reaction. As described in the text, the information content of the enzyme is expressed geometrically by the formation of a shape within the protein that is precisely complementary to the shape of the substrate. The information is, thus, converted to energy by reducing Ea (ΔEa).

By this effect, the enzymes are catalysts that do not alter the fundamental thermodynamics of the reaction, in the sense that the initial thermodynamic state of substrate and the final thermodynamic state of the products are not changed [8]. Because it acts as a catalyst, the enzyme is not consumed in the reaction so that its information content is applied repeatedly provided substrate is available and no additional post-translation modifications occur. Typically enzymes accelerate reactions, often by many orders of magnitude (Fig 1). Without them, many reactions—e.g., reactions to extract energy from substrate or synthesize cell components—would be too slow to permit orderly function of living systems. We propose that this characteristic of enzymes permits investigation of the relation of information to thermodynamics and order through the concept of “activation energy.” Finally, we note recent studies [8,9] have emphasized the dynamic nature of enzyme structure and the critical role of structural motion of the protein during catalysis. By integrating these dynamics into our model, we note that quantum effects may be observed.

## Modeling Methods and Results

### Key-Lock dynamics

**Fig 2:**
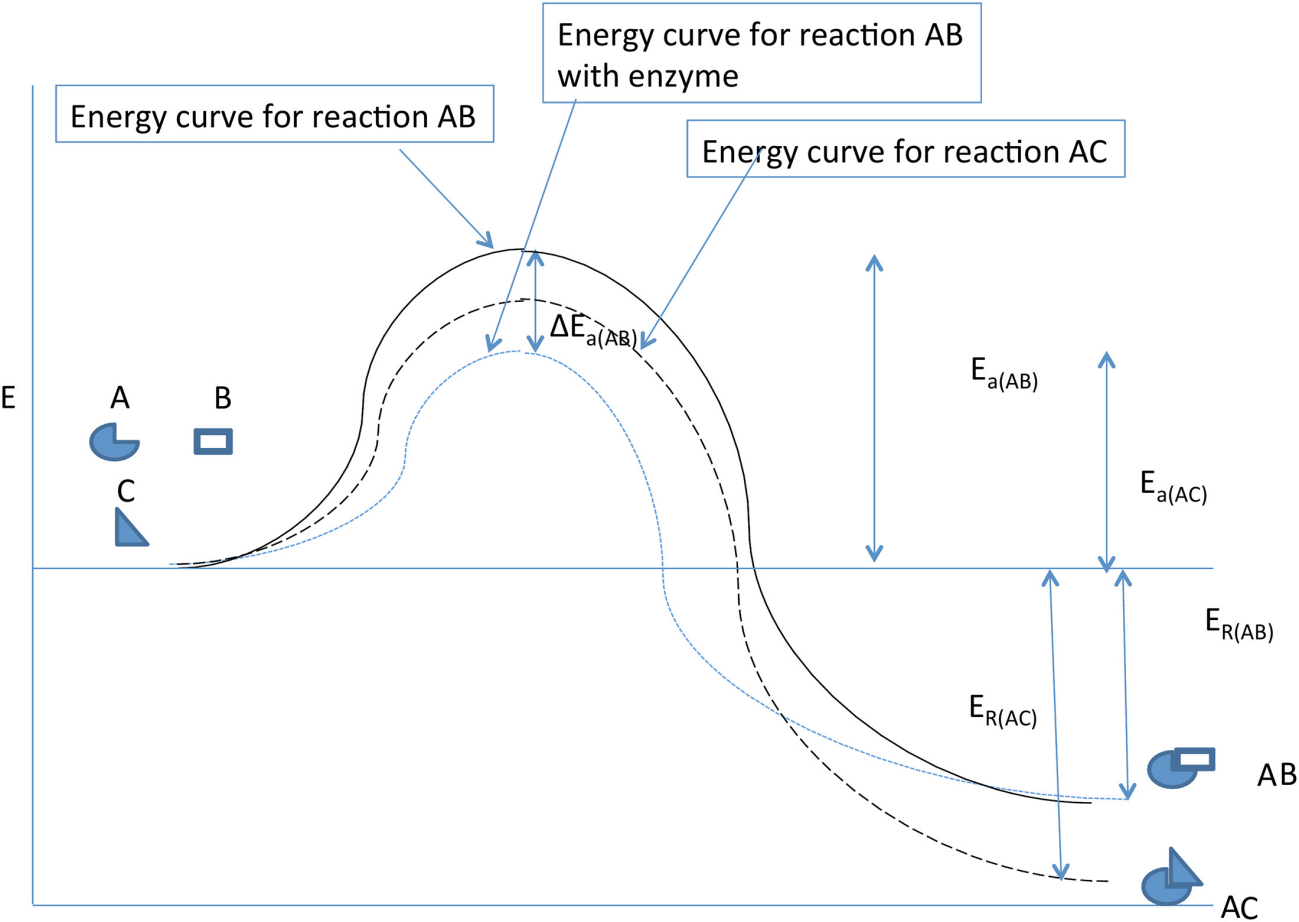
Information in living systems manifest through “temporal gradients”. Here the system contains initially two substrates and one enzyme. In the absence of the enzyme, reaction C → G + H will proceed more rapidly because it has both lower final free energy and lower activation energy. However, the enzyme lowers the Ea for reaction B → E + F. The information in the enzyme produces an observable gradient over time as the concentrations of E and F are increased and B is decreased when compared to an uncatalyzed system. In contrast, because of its specificity, the enzyme has no effect on the temporal evolution of the substrate and product concentrations of reaction C → G + H.

Enzymes are typically highly specific, decreasing the activation energy *(E*_*a*_) (Figs 1 and 2) and increasing the reaction rate (*k*) only for a small number of substrate molecules [9]. This link between *E*_*a*_ and *k* is typically described by the empirically derived Arrhenius equation (see below). The specific activity of the enzyme is often described as a “lock and key” [10] process in which some region of the folded protein provides a complementary geometric shape to that of the substrate [11,12] thus reducing the entropy of the interactions. We note that enthalpic interactions such as Coulomb interactions are also maximized as the distance between substrate and enzyme is decreased where, as noted in [12], “interactive enthalpy is estimated from the sum of electrostatic and van-der-Waals interactions.” This permits binding that facilitates the reaction often through complex intermediate transitory steps.

Here we will focus on the spatial interactions between enzyme and substrate. We will view the catalyzed reaction as a single step of substrate → products (Figs 1 and 2) omitting for simplicity the transient intermediate steps. We initially assume an enzyme density law *r*_*n*_ = *r*(*x*_*n*_), *n* = 1,…,*N* with the proteins fixed at molecular positions *x*_*n*_ = *n*Δ*x*, *n* = 1,…,*N*. For simplicity, a one dimensional case is temporarily assumed, and with constant position spacings Δ*x*. These constraints will be relaxed in subsequent sections.

Let the substrate pathway positions obey an unknown density law *p*(*x*_*n*_), *n* = 1,…,*N* on the pairs of substrate particles that ordinarily constitute reactant molecules. Let these reactant molecules interact, or ‘bind,’ with the enzyme molecules. This defines an enzyme-substrate complex.

It is shown (see Appendix) that this complex lowers the activation energy of the reaction. One of the most important ways that an enzyme catalyzes any given reaction is through entropy reduction: by bringing order to a disordered system. Thus, since entropy is a component of Gibbs free energy, this free energy is lowered as well. This in turn is a component of the activation energy *E*_*a*_ which, as mentioned above, is likewise lowered. These factors work to increase the reaction rate. They also accelerate the reaction by providing a spatially specific charge distribution that form bonds with substrate to accelerate the reaction process. Enzymes also promote chemical reactions by bringing substrates together in an optimal orientation, lining up the atoms and bonds of one molecule with the atoms and bonds of the other molecule. This constitutes a lowering of local entropy, in particular the Kullback-Leibler or ‘cross’ entropy (as will be seen).

The initial interaction between enzyme and substrate is relatively weak, but these weak interactions rapidly induce conformational changes in the enzyme that strengthen binding [13]. These conformational changes are augmented by a ‘key and lock’ effect whereby the substrate ‘key’ molecule fits *optimally close* to the complementary 3 dimensional structure within the enzyme ‘lock’ particle. This ‘key/lock’ effect tends to maximize the reaction rate.

Initially assuming a well-mixed distribution of enzymes and substrate of equal concentration, we view the “lock” as constantly-spaced enzyme molecules of density profile *r*_*n*_ = *r*(*x*_*n*_ + Δ*x*/2), *x*_*n*_ = *n*Δ*x*, Δ*x* small. These molecules are located at positions (*n* + 1/2)Δ*x* with density values *r*_*n*_. And by comparison, the substrate (or “key”) molecules are particle pairs having a local density profile *p*_*n*_ = *p*(*x*_*n*_) at positions *x*_*n*_ = *n*Δ*x*. These are thereby located *halfway between* corresponding lock molecules *r*_*n*_. Each enzyme-substrate ‘complex’ locally lowers the activation energy of the reaction so that overall activation energy is maximally lowered when all key particles are ‘closest’ geometrically to the corresponding lock particles.

This is exemplified in Figs 1 and 2.

Then, given a fixed enzyme path *r*_*n*_, *the problem of minimizing activation energy becomes one of geometry*. *What substrate reactant path p*_*n*_ *obeys minimal distance from the fixed enzyme path r*(*x*_*n*_)?

#### Kullback-Leibler measure

We now need to choose a measure of the distance between the two density paths. From the preceding, this distance is to be a minimum. One useful measure is their Kullback-Leibler [14,15] ‘divergence,’ defined as

$$H_{KL}(p||r\text{)}=\sum_{n=1}^{N}p_{n}\text{ln}\left[\frac{p_{n}}{r_{n}}\right].$$ (1a)

Although *H*_*KL*_ is not formally a ‘distance’ (since it is not symmetric in *p* and *r*) it has many properties of one and, for our purposes, is convenient to be regarded as such. It also obviously has the form of an ‘entropy,’ and so can be termed ‘KL entropy’.

The KL distance between all enzymes of density *r*_*n*_ = *r*(*x*_*n*_ + Δ*x*/2) and their corresponding substrate molecules of density *p*_*n*_ = *p*(*x*_*n*_) is to be minimized, obeying

$$H_{KL}(p||r\text{)}=min.$$ (1b)

We are here analyzing a one-dimensional problem, i.e. where each *x*_*n*_ and Δ*x* is a scalar value. But this ignores the vital question of *relative orientation* of key and lock molecules. That is taken up at the end, and is an easy generalization of the one-dimensional approach.

This geometrical interleaving of the two types of molecule does represent a one-dimensional form of a key-lock geometry. However, specifically what *density function p*(*x*_*n*_) should govern the reactant pathway?

#### Derivation of optimum reactant pathway *p*_*n*_

Regarding *all* enzyme and reactant molecules, this is assumed to obey principle (Eq 1a and Eq 1b). The reactant is also the substrate, so we are seeking the substrate *density* function *p*_*n*_ that has minimum KL distance from the *given* enzyme pathway *r*_*n*_, *n* = 1,…,*N*. This is assumed to occur in the presence of the interlacing (*x*_*n*_, *x*_*n*_ + Δ*x*/2) of coordinate positions defined above, and also the known physical constraints of the problem. The main one is that of known mean energy.

We seek the pathway position law *p*_*n*_ that obeys *H*_*KL*_(*p*||*r*) = *min*., in the presence of the arbitrary, but fixed, enzyme pathway *r*_*n*_. (Note: This temporarily ignores the more recently observed effect of “induced fit [16,17],” whereby the enzyme pathway changes as well to further improve the fit. This is addressed below. The two laws *p*_*n*_, *r*_*n*_ of course obey normalization

$$\Sigma_{n}p_{n}=1,\;\Sigma_{n}r_{n}=1,\;\;r_{n}=const.,\;n=1,\ldots ,N.$$ (2)

(All sums are over the entire pathways.). Assume, as well, a fixed, *mean* molecular bond energy

$$\Sigma_{n}P(E_{n})E_{n}=\;\Sigma_{n}p_{n}E_{n}=\;kT,\text{ with }P(E_{n})=\;p_{n}$$ (3)

by definition, *κ* Boltzmann’s constant and *T* a fixed energy. Energies *E*_*n*_ could, e.g., be due to hydrogen bonds. Also, Eq (3) assumes ergodicity to hold. That is, the true statistical average energy—the left-hand sum—equals the average energy along any one path—the second sum. We will use this ergodic property below.

#### Net Optimization Problem

We therefore seek the reaction (or substrate) rate *p*_*n*_ satisfying KL requirement Eq (1b) subject to four constraints Eqs (2) and (3) obeyed by *p*_*n*_ and *r*_*n*_. By the method of undetermined multipliers, these satisfy the variational principle

$${\text{Σ}}_{n}p_{n}\text{ln }\left[\frac{p_{n}}{r_{n}}\right]\;+\wedge_{1}\left[\Sigma \_p_{n}\;-\;1\right]+\;\wedge_{2}\left[\Sigma \_r_{n}\;-\;1\right]\;+\;+\;\wedge_{3}\left[\Sigma \_p_{n}E_{n}-\kappa \text{T}\right]=min.$$ (4)

Differentiating this ∂/∂*p*_*n*_ and equating it to zero gives as the condition for the constrained minimum

$$1+lnp_{n}-lnr_{n}+\wedge_{1}+\wedge_{3}E_{n}=0.$$ (5)

Solving Eq (5)

$$\;p_{n}=r_{n}\;exp\left[-1-\wedge_{1}-\wedge_{3}E_{n}\right]$$ (6)

On this basis, for a given point *n*, the maximum probable local reaction rate *p*_*n*_ ≡ *p*(*x*_*n*_) is proportional to the neighboring (at positions *x*_*n*_ ± Δ*x*/2) densities *r*_*n*_ of the enzyme. This makes sense since each enzyme is assumed to locally enhance the reaction, e.g. by strong hydrogen bonding, and this enhancement becomes stronger the geometrically closer the reactant is to the enzyme.

The rate *p*_*n*_ of reaction in Eq (6) also falls off with the local molecular bonding energy *E*_*n*_. This also makes sense since the stronger the bond is the less probable it is that the molecule breaks up and contributes to the desired reactant.

#### Derivation for Multi-dimensional Geometry

For optimum key-lock fit, the two molecules must not only be optimally close but also each have a correct orientation. The approach to this problem requires a generalization to the use three-dimensional variables ***x***_*n*_ ≡ (*x*,*y*,*z*)_*n*_. Here *p*_*n*_ = *p*(*x*,*y*,*z*)_*n*_, etc. for *r*_*n*_ and with Δ*x*→Δ***x*** = (Δ*x*,Δ*y*,Δ*z*)_*n*_. Also, the Kullback-Leibler distance is of the same form Eq (1a) as before,

$$\Sigma_{n=1}^{N}p\left(x_{n}\right)\text{ln}\left[\frac{p\left(x_{n}\right)}{p\left(x_{n}+\Delta x/2\right)}\right]=min.$$ (7)

The identical algebra Eqs (3)–(6) follow as before, with boldface quantities replacing scalars, but with the scalar *E*_*n*_ remaining in Eq (6) since energy is always a scalar quantity. However, an important new interpretation arises for the effect Δ***x*** → **0**. Acknowledging this to occur in three dimensions requires the key and lock to now approach one another while in *the same orientation*. This describes a true key-lock bond. Also, now the change of reactant path so as to reduce activation energy *E*_*a*_ occurs in full three-dimensional space.

Note that principle Eq (7) is much more than simply a 3D version of principle (Eq 1a and Eq 1b). Consider the 3D tissue produced by multiply-folding a long string of nucleotides. From the form of Eq (7), the more regular the folding is, i.e. the more often *p* a given codon occurs at *neighboring* points ***x***_*n*_, the closer to 1 will be the ratios in the logarithm ln operation in Eq (7). Therefore the smaller will be their contributions to Eq (7) after the ln is taken. Hence the smaller will be the minimum value of *H*_*KL*_. Tissue with such low cross-entropy has low free energy and a high state of order. This might account for the vital role played by protein folding in augmenting living systems [6]. In turn, this emphasizes that *H*_*KL*_ has direct biological significance as a measure of cellular growth, despite being merely a geometrical measure.

#### Deriving the Arrhenius equation

The Arrhenius equation describes the dependence of reaction rates upon temperature and is empirically-derived. No enzymes are presumed present. Or equivalently, they are *equally* present at all reaction path positions [18]. Hence, we now repeat use of the minimum Kullback-Leibler principle in the special case where all enzyme densities obey

$$\;\;r_{n}=r=const.$$ (8)

Also, for simplicity we return to the one dimensional case of scalar coordinates *x*_*n*_. Recall that we used the ‘ergodic hypothesis,’ that the statistics of *E* at any position *x*_*n*_ equals that of *E* over *any one path x*_*n*_, *n* = 1,…,*N*. On this basis, and using the last identity Eq (3), result Eq (6) is, in the special case Eq (8)

$$P\left(E_{n}\right)=Kexp[-\wedge_{3}E_{n}],\;\;K=\;rexp\left[-1-\wedge_{1}\right].$$ (9)

We also found, at Eq (3), that the average < *E* > = *kT*. Using this in Eq (9) gives = *∧*_3_ = *K* = 1/*kT*. Then

$$P\left(E_{n}\right)={\left(\kappa T\right)}^{-1}exp[-E_{n}/\kappa T],$$ (10)

the Boltzmann energy distribution law.

At this point it is assumed that if the energy *E*_*n*_ ≥ *E*_*a*_, a so-called ‘activation’ level of the energy, the reaction occurs at the position *x*_*n*_. But we also assumed ergodicity to hold. Therefore, the reaction occurs as often as event *E*_*n*_ ≥ *E*_*a*_ occurs for any one *n*. This shows that for any fixed energy density function *p*(*E*_*n*_) the smaller *E*_*a*_ is the more energy events *E*_*n*_ occur or, equivalently, the higher is the reaction rate.

Also, ergodicity allows us to now drop subscript *n* in Eq (10). Then using Eq (10) for *p*(*E*) gives

$$P\left(E\geq E_{a}\right)=\int_{E_{a}}^{\infty }dE\;P\left(E\right)={\left(\kappa T\right)}^{-1}\int_{E_{a}}^{\infty }dE\text{ exp}\left(-E/\kappa T\right)\;=\;exp\left(-\frac{E_{a}}{\kappa T}\right).$$ (11)

Since each energy value *E* satisfying Eq (11) gives rise to a reaction product, this shows that the reaction rate grows as the activation energy *E*_*a*_ decreases.

But the analysis has ignored the fact that the molecules of the reacting medium may have a known *prior* probability *A* of being in the proper orientation to react. This probability should multiply result Eq (11).

The result is that the net probability density, or reaction rate, obeys

$$k=\;AP\left(E\geq E_{a}\right)=A\;\;exp\left(-\frac{E_{a}}{\kappa T}\right),$$ (12)

The Arrhenius equation.

As we discussed, the optimum choice of enzyme path *r*_*n*_ for accomplishing the desired reaction can occur along an altered reaction path *x*_*n*_ requiring *a lower* activation energy *E*_*a*_. This is shown by Eq (12) in two ways:, First, the required energy values *E* can be smaller; and second, the resulting reaction rate *k* is higher. That *E*_*a*_ is, in fact, a minimum is shown in the Appendix *to follow* from the *H*_*KL*_ principle (Eq 1a and Eq 1b). Thus, the *H*_*KL*_ principle derives both the well-known rate effect Eq (12) and the fact that activation energy *E*_*a*_ tends to be a minimum value.

### Optimization of reactant path by quantum entanglement

In the preceding, only densities *p*_*n*_ were optimized for a fixed enzyme density path *r*_*n*_. However, further optimization can be made whereby the *r*_*n*_ themselves are allowed to change so as to further improve the key/lock fit. This is called “conformer selection” or “induced fit.”[18]. We propose two effects that potentially accomplishing this.

As noted above, enzyme function requires a tight geometric fit in which the atoms of the amino acids in to protein and the substrate molecules are separated by distances that are minimized. Suppose, as we found, their spacings Δ*x*/2 are on the order of angstroms. At such molecular distances, quantum effects can enter in, e.g. in the form of quantum *entanglement*. This is even for semiclassical quantum effects [19]

Other authors [20], in fact, define the degree of *global entanglement* between two systems *p*_*n*_, *r*_*n*_ as the very value of *H*_*KL*_(*p*||*r*) for the *p*_*n*_, *r*_*n*_ obeying KL principle (Eq 1a and Eq 1b). That is: *The level of entanglement is defined by the minimized value of the Kullback-Leibler entropy*, *which was our very criterion* (Eq 1a and Eq 1b) *for the choice of the p*_*n*_.

This also makes intuitive sense: By Eq (1a) ‘distance’ measure *H*_*KL*_(*p*||*r*) is mathematically at its absolute minimum value, of zero, when all *p*_*n*_ = *r*_*n*_. This describes perfect entanglement between the the two systems *p*_*n*_, *r*_*n*_, so that interchanges

$$p_{n}⇆r_{n}$$ (13)

of the roles played by enzymes and reactants repeatedly take place. By the same token, finite values, instead, of *H*_*KL*_(*p*||*r*) allow only certain pairs of the *p*_*n*_, *r*_*n*_ to effectively interchange roles. It results, then, that over a number of such reactions the initial molecular reactant paths *p*(*x*_*n*_), *r*(*x*_*n*_), *n* = 1,…,*N* can progressively wander off to totally different ones which further upgrade the key/lock fit. These are also, in fact, *energetically preferred* since, by Eq (12), the progressively lowered threshold energy *E*_*a*_ is more readily provided at each such entanglement.

## Discussion

Here we investigate a mechanism by which living systems use information to maintain a low entropy state far from thermodynamic equilibrium. We propose that the information encoded in the inherited sequence of nucleotides in DNA is manifested geometrically in the 3 dimensional shape of an enzyme determined by the lowest free energy state of the amino acid sequence specified by the corresponding gene. However, we note that the 3 dimensional shape of the enzyme can be extensively altered by post-translation modified. Thus, the geometry of the enzyme represents a summation of heritable information represented by its amino acid sequence and temporally variable information regarding the state of the cell and its environment which govern post translation modification.

Most simply, the information within the 3 geometry of the protein is manifested thermodynamically by the reduction in the activation energy (*E*_*a*_) of the reaction catalyzed by the enzyme.

The mechanism by which information reduces the activation energy is geometric as, like a “lock and key”, the shape of the enzyme precisely fits the shape of a substrate. We investigate these spatial interactions using the Kullback-Leibler distance, Eq (1a), which is a generalization of the Shannon mutual information. In fact in many textbooks the latter is derived as a special case of the former. We demonstrate that the information of the enzyme “lock” vis a vis the shape of the substrate “key” is the equivalent of the K-L distance. Maximum information corresponds to a minimal K-L distance and, thus, the largest possible decrease in the *E*_*a*_.

The observable effect of the enzyme-induced decrease in *E*_*a*_ is an increase in the reaction rate *k*, often by several orders of magnitude. This is quantified by the empirically-derived Arrhenius equation. Here we demonstrate that the Arrhenius equation can be derived from a first principle that requires minimum Kullback-Leibler divergence, (Eq 1a and Eq 1b), between a *fixed* enzyme density function and an unknown reactant function.

Here we also investigate the more recently proposed “induced fit” model in which the enzyme geometry *changes* in response to the substrate thus further improving the geometric match. Interestingly, we find that the induced fit dynamic will occur over very small molecular distances Δ*x*, which will potentially permit quantum entanglement effects. In particular, we find for small Δ*x the minimized KL entropy becomes proportional to the degree of quantum entanglement* of path functions *p*_*n*_, *r*_*n*_. This extends prior studies suggesting quantum effects in proteins including enzymes [21–23].

Our investigation also provides general insights into the dynamics of biological information. Although it is clear that information must play a central role in the growth of living systems, the general principles that govern translation of information into biological order and function are not well defined [24]. We note that an enzyme can alter the living system in ways similar to the classic Maxwell’s demon *gedanken [25,26]*. For example, a protein within a membrane can use its information (expressed as its 3 dimensional shape) to select and bind a substrate on one side of the membrane and move it into the adjacent cellular compartment [27] thus creating a spatial concentration gradient similar to the classic thought experiment [28]. However, unlike the iconic demon, enzymes can also generate a gradient *over time [29]*. That is, by greatly accelerating the rate of reaction, the concentration of substrate and products over time will be larger and smaller respectively when an enzyme is present compared to a system in which the information content of the enzyme is absent.

Finally, we note that biological information in our study is highly contextual. This is apparent, in Figs 1 and 2, as an enzyme-dependent quantitative change in activation energy *E*_*a*_ is dependent on both the properties of the enzyme and the properties of the substrate. Thus, in Fig 2, addition of an enzyme that is specific to the AB reaction, but not the AC reaction, lowers *E*_*a*_ for the AB reaction relative to that for the AC. As a result the energy *E* of system AB will much more often obey *E* ≤ *E*_*a*_ and, hence, occur much more often than the reaction AC. The information in the enzyme can, thus, be viewed as “kinetic” in reaction AB and only “potential” in the absence of the substrate. Restating this quantitatively, the information of an enzyme is defined by the KL divergence between the enzyme and a potential reactant. Further, the level of this information in each biological enzyme is converted to a thermodynamic property by the change in *E*_*a*_ that it evokes. Thus, the information may be either ‘potential’ or ‘kinetic,’ depending on context. The kinetic information represents the increased probability of a reaction and decreased *E*_*a*_, when substrate to which it can bind is presence according to principle (Eq 1a and Eq 1b). By contrast, the same enzyme but in the presence of substrate with which it *cannot react* (or in the absence of substrate) carries only potential information. It is interesting that such contextual dependence is lacking in, e.g., the pure Shannon entropy [30] measure *H*_*S*_ = −∫*p*(*x*)*lnp*(*x*). The algebraic difference is that the KL information is of *p* in the presence of context *r* whereas the Shannon *H*_*S*_ is in *p* by itself, *in the absence of* any context *r*. In summary, it is the contextual dependence of the KL information that provides its biological significance and gives rise to its function.

## Appendix

### The *H*_*KL*_ principle implies as well that activation energy *E*_*a*_ = *minimum*

Enzymes perform the critical task of lowering the activation energies *E*_*a*_ of chemical reactions inside the cell. For example, it is obvious from the form of the rate Eq (12) that the reaction rate *k* is maximized when energy *E*_*a*_ is a minimum value. But specifically *what effect* lowers *E*_*a*_? Could it, e.g., be our working principle Eq (1b) *H*_*KL*_(*p*||*r*) = *minimum*? If so, the principle would now have doubled value. This is verified next.

It is convenient to work with the continuous version of *H*_*KL*_ Eq (1a), where the general coordinates *x*_*n*_ go over into continuous energy values *E*_*n*_ = *E*. Also use the route (setting *r*_*n*_ = *r* = *const*.) Eq (8) to the Arrhenius equation. Then the principle Eq (1b) becomes

$$H_{KL}={\int^{\;}}^{\;}dEP(E)ln\left(\frac{\_P\left(E\right)}{r}\right)=min.$$ (A1)

where by Eq (10)

$$P\left(E\right)={\left(\kappa T\right)}^{-1}\text{exp}\left(-\frac{E}{\kappa T}\right).$$ (A2)

Expanding the *ln* in principle Eq (A1) gives directly

$$H_{KL}={\int^{\;}}^{\;}dEP(E)lnP\left(E\right)-\;\;\text{ln}\left(r\right){\int^{\;}}^{\;}dEP(E)=min.$$ (A3)

Using expression Eq (A2) for *P*(*E*) in Eq (A3), and the normalization of *P*(*E*), give

$$H_{KL}=\;\int_{E_{a}}^{\infty }dE\text{ exp}\left(-\frac{E}{\kappa T}\right)[-\left(\frac{E}{\kappa T}\right)-ln\left(\kappa T\right)]-\left(\kappa T\right)\text{ln}\left(r\right)=min.$$ (A4)

Why is *E*_*a*_ the lower integration limit? Since our aim centers on the value of rate *k* we only integrate over those values of *E* that can contribute to *k*, and by Eq (12) this is the value *E*_*a*_.

Dividing through Eq (A4) by *kT* and doing the integrations gives a condition

$$\frac{H_{KL}}{\kappa T}\equiv y=\text{exp}\left(-\frac{E_{a}}{\kappa T}\right)\left(\frac{E_{a}}{\kappa T}+1\right)-\text{ln}\left(\kappa T\right)-\text{ln}\left(r\right)\equiv min.$$ (A5)

To attain the required minimum in *H*_*KL*_ through choice of *E*_*a*_ requires setting $\frac{\partial y}{\partial E_{a}}=0.$ Differentiating Eq (A5) in this way gives a requirement

$$\frac{E_{a}}{\kappa T}\text{exp}\left(-\frac{E_{a}}{\kappa T}\right)=0.$$ (A6)

This is accomplished by either *E*_*a*_ = 0 or *E*_*a*_ = *∞*. From the result Eq (12) for the reaction rate *k* it is obvious that these activation energy values respectively maximize, or minimize, the rate *k*. Of course the case *E*_*a*_ = 0 is preferred on the basis of maximum reaction rate. However, our aim here is to show that this activation energy also follows from our overall principle (Eq 1a and Eq 1b) that *H*_*KL*_ = *min*. Since Eq (A5) gives *H*_*KL*_ (proportional to *y*) we can use it to judge if the usual requirement for attaining a minimum is satisfied, namely that the second derivative $\partial^{2}y/\partial E_{a}{}^{2}>0.$ Taking this second derivative gives the anticipated result

$$\frac{\partial^{2}y}{\partial E_{a}{}^{2}}=\frac{1}{{\left(\kappa T\right)}^{2}}\;>\;0.$$ (A7)

Hence the case *E*_*a*_ = 0 both maximizes the reaction rate *k* and minimizes *H*_*KL*_ as required. By Eq (A5) zero activation energy gives a minimum *H*_*KL*_ of value

$$H_{KL}\;=\kappa T\left(1-ln\left(\kappa rT\right)\right).$$ (A8)

Of course attaining activation energy *E*_*a*_ = 0 is not a usual case, but the analysis shows that the closer the system gets to achieving it the higher the reaction rate is, and the smaller the KL distance is between enzyme and substrate, i.e. the better does the key fit into the lock.
